# What is the purpose of ultra-processed food? An exploratory analysis of the financialisation of ultra-processed food corporations and implications for public health

**DOI:** 10.1186/s12992-023-00990-1

**Published:** 2023-11-13

**Authors:** Benjamin Wood, Ella Robinson, Phillip Baker, Guillermo Paraje, Mélissa Mialon, Christoffer van Tulleken, Gary Sacks

**Affiliations:** 1 Global Centre for Preventive Health and Nutrition, Institute for Health Transformation, Deakin University, Geelong, Australia; 2 Institute for Physical Activity and Nutrition, Deakin University, Geelong, Australia; 3 Business School, Universidad Adolfo Ibañez, Santiago de Chile, Chile; 4 Trinity Business School, Trinity College, Dublin, Ireland; 5 University College London, London, UK

**Keywords:** Ultra-processed foods, Dietary transition, Financialization, Corporate governance, Commercial determinants of health

## Abstract

**Background:**

In recent decades there has been a global rise in consumption of ultra-processed foods (UPFs) to the detriment of population health and the environment. Large corporations that have focused heavily on low-cost manufacturing and extensive marketing of UPFs to maximise profits have driven this dietary transition. The same corporations claim to serve the interests of multiple ‘stakeholders’, and that they are contributing to sustainable development. This paper aimed to test these claims by examining the degree to which UPF corporations have become ‘financialised’, focusing on the extent to which they have prioritised the financial interests of their shareholders relative to other actors, as well as the role that various types of investors have played in influencing their governance. Findings were used to inform discussion on policy responses to improve the healthiness of population diets.

**Methods:**

We adopted an exploratory research design using multiple methods. We conducted quantitative analysis of the financial data of U.S. listed food and agricultural corporations between 1962 and 2021, share ownership data of a selection of UPF corporations, and proxy voting data of a selection of investors between 2012 and 2022. We also conducted targeted narrative reviews using structured and branching searches of academic and grey literature.

**Results:**

Since the 1980s, corporations that depend heavily on manufacturing and marketing UPFs to generate profits have been increasingly transferring money to their shareholders relative to their total revenue, and at a level considerably higher than other food and agricultural sectors. In recent years, large hedge fund managers have had a substantial influence on the governance of major UPF corporations in their pursuit of maximising *short-term* returns. In comparison, shareholders seeking to take steps to improve population diets have had limited influence, in part because large asset managers mostly oppose public health-related shareholder proposals.

**Conclusions:**

The operationalisation of ‘shareholder primacy’ by major UPF corporations has driven inequity and undermines their claims that they are creating ‘value’ for diverse actors. Measures that protect population diets and food systems from the extractive forces of financialisation are likely needed as part of efforts to improve the healthiness of population diets.

**Supplementary Information:**

The online version contains supplementary material available at 10.1186/s12992-023-00990-1.

## Background

The rapidly growing share of ultra-processed foods (UPFs) in human diets raises serious concerns for public and planetary health [1, 2]. According to NOVA, a widely recognised food categorising system used in multiple national dietary guidelines [3, 4], UPFs are ‘*formulations of ingredients, mostly of exclusive industrial use, that result from a series of industrial processes*’ [5]. Common examples of UPFs include carbonated soft drinks, industrially made snacks and breads, reconstituted meat products, ice creams, confectionery, and many types of breakfast cereals [5]. UPFs are distinct from fresh and ‘processed’ foods, which include tinned, frozen, and cooked foods made using ingredients commonly found in domestic kitchens around the world.

There is a substantial and growing evidence base linking high levels of consumption of UPFs with adverse population health outcomes, including all-cause mortality, overweight and obesity, chronic diseases (e.g., heart disease, type-2 diabetes, cancer, dementia and depression), and poor pregnancy and childhood developmental outcomes [6–14]. In addition, UPF production and consumption are associated with a range of poor environmental outcomes, including high levels of plastic waste and pollution, the monocultural production of commodity crops and related impacts on biodiversity [15–18].

Unlike other foods, UPFs are exclusively made by for-profit corporations, and it is well recognised that these corporations have played a large role in driving the global UPF dietary transition [19–22]. Since the advent of ultra-processing technologies in the late 19th century, ultra-processing has become a core strategy of profit maximisation within the industrial food system [23–25]. The competitive and financial advantages that ultra-processing confers food corporations, such as by increasing product durability, as well as by facilitating product and brand differentiation, is likely one of the key explanatory factors as to why today’s major UPF corporations have been able to accrue extensive resources and capacities over a long period of time [22]. Such resources and capacities have, in turn, underpinned the deployment of diverse strategies (e.g., global expansion, aggressive marketing, lobbying) by major UPF corporations intended to shape population diets and food systems in their favour [19, 26, 27].

Corporate strategy alone, however, does not explain the global rise of UPFs. In recent years, a growing body of work has examined the ways in which various widespread and interlinked processes have facilitated the global UPF dietary transition by shifting institutional and governance arrangements towards accommodating, rather than constraining, the power and legitimacy of UPF corporations [19, 20]. Such processes include, inter alia, the industrialisation of food systems, economic globalisation, trade and investment liberalisation, and increases in multi-stakeholderism. While these processes influence countries and social groups in different ways, exploring their role in driving the global UPF dietary transition can help to shed light on various structural and transformative changes with the potential to curb the global UPF dietary transition [20, 28].

A key capitalist process that relates to, and in some ways underpins, many of the above-mentioned processes is ‘financialisation’. Epstein (2005) refers to financialisation as the ‘*increasing role of financial motives, financial markets, financial actors and financial institutions’* in the economy [29]. Others have described financialisation as the methods and practices through which value is increasingly extracted from the ‘real economy’ (i.e., the section of the economy concerned with the production, trade and consumption or use of goods and services) into the ‘financial economy’ (i.e., the section of the economy that solely deals in transactions involving money and other financial assets) [30–32]. Despite financialisation being one of the defining features of modern food systems [33, 34], and contemporary capitalism more broadly [29–32], there has only been limited focus in the public health literature on the relationship between financialisation and the global rise of UPFs.

In this paper, we focus on one key aspect of financialisation that has been described as a potentially important shaper of population diets and food systems: the increasing prioritisation of the financial interests of shareholders and owners by the decision-makers of UPF corporations above other economic, health, social and ecological considerations [22, 35]. The norm underpinning this form of corporate governance is often labelled ‘shareholder primacy’, referring to the belief or view that the sole purpose of the business corporation should be to maximise financial returns for its shareholders or private owners [36]. Various neoliberal and neoclassical assumptions are often used to legitimise and justify this form of corporate governance, often by contending that it is the most rational and efficient way of achieving the broader social good [36]. While ‘shareholder primacy’ has emerged to dominate the theory and practice of corporate governance in the world economy, sector-specific analysis can help to determine the extent to which the norm has been operationalised by a particular sector, as well as expose some of its sector-specific implications [36, 37].

We argue that examining the extent to which ‘shareholder primacy’ influences the governance of UPF corporations is important for several reasons. To start with, this type of examination can help to scrutinise many of the responsibility and sustainability claims made by UPF corporations. Such claims include ‘creating shared value’ in order to ‘enhance quality of life for everyone’ [38], supporting communities to build a ‘better shared future’ [39], and building sustainable economies [40]. There are growing concerns that these claims are an attempt by UPF corporations to position themselves as ‘part of the solution’ to the problems they perpetuate [41]. This positioning, in turn, seeks to legitimise corporate and industry self-regulation, as well as the participation of major UPF corporations in national and international governance arrangements ostensibly designed to address diet-related social and environmental harms [19]. More broadly, these claims can be understood as part of narratives on ‘stakeholder capitalism’ championed by various prominent pro-corporate organisations [42, 43]. As a case in point, in 2020, the World Economic Forum’s Annual Meeting focused on renewing the concept of ‘stakeholder capitalism’, which the organisation defined as a ‘form of capitalism in which companies seek long-term value creation by taking into account the needs of all their stakeholders, and society at large’ [44], as a means of ‘overcom[ing] income inequality, societal division, and the climate crisis’ [45].

An examination of the governance of UPF corporations also encourages analysis of the ways in which different types of investors influence the behaviour of these corporations, a topic that has received minimal analytical attention in the public health literature. While most investors typically strive to maximise their returns on the investments they make, their approaches and perspectives vary, as does their influence on corporate governance [46]. For instance, interest from so-called ‘responsible investors’ in improving population diets is reportedly building, often as part of broader environmental, social and governance (ESG) initiatives [47]. Responsible investors often try to influence corporate governance via several complementary mechanisms, including by filing or supporting shareholder proposals, i.e., a proposed recommendation or requirement that the corporation or its board of directors take a specific course of action that is put to vote at shareholder meetings [47].

At the same time, campaigns from investors seeking to maximise their returns in the *short-term* are common and widespread [46]. In particular, it has been noted that so-called hedge fund ‘activists’ have played a major role in reinforcing ‘shareholder primacy’, especially since the Global Financial Crisis [46]. One of the core investment strategies of hedge fund ‘activists’ involves purchasing a minority stake in a publicly listed corporation in order to influence the way in which it is governed [48]. Specifically, many hedge fund ‘activists’ seek to maximise shareholder returns in the short-term by pressuring corporate decision-makers to undertake large-scale cost-cutting practices (e.g., large job cuts) and to increase shareholder payouts [46]. In this way, hedge fund activism is typically distinctly different from activism oriented towards public health objectives, and social and ecological justice more broadly. Strategies that have been used by hedge fund ‘activists’ to directly influence corporate governance include attaining representation on corporate boards, working with other shareholders to gather enough votes to overthrow corporate board members, and publicly applying pressure on corporate decision-makers to meet their demands [48, 49].

Large asset management firms, such as BlackRock and Vanguard, are another important type of investor that warrant further public health attention. Research has shown that a relatively small number of asset management firms now hold a large proportion of shares across the entire corporate food system and, more broadly, the corporate economy [50, 51]. As such, they are conferred with substantial powers to shape corporate governance, including with respect to their outsized influence on the outcome of shareholder proposals [51]. While evidence suggests that many of the world’s largest asset management firms vote against shareholder proposals that seek corporate action on various social and environmental issues [52], the extent to which they oppose public health-related shareholder proposals that target major UPF corporations remains unclear.

Given the above considerations, this paper aimed to explore the degree to which UPF corporations have become ‘financialised’, focusing on the extent to which they have been prioritising the financial interests of their shareholders relative to other actors, as well as the role that various types of investors have played in influencing their governance. Specifically, the paper had three related objectives. First, to document and describe trends in the monetary value of shareholder wealth and income generated by UPF corporations relative to corporations active in other food and agricultural sectors. Second, to document and describe the extent to which the world’s major UPF corporations have been prioritising the *short-term* financial interests of their shareholders. Third, to explore some of the ways in which various types of investors – notably, responsible investors, hedge fund activists, and large asset managers – have influenced the governance of the world’s major UPF corporations. The findings were used to inform discussion on potential challenges and opportunities for advocates, researchers, and policy-makers seeking to address the global rise in the consumption of UPFs.

## Methods

### Overview of research design

We adopted an exploratory research design using multiple methods. Drawing from approaches described elsewhere [22, 51], we used a range of methods to address the aims and objectives of this paper (see Table 1). Data used for quantitative analysis, including company financial, share ownership and proxy voting data, were sourced from several databases (described below). Periods of quantitative analysis were based on available data and practical considerations. To complement findings from the quantitative analyses (e.g., by providing historical context) and to examine additional information gaps (e.g., in relation to hedge fund activism), we also conducted a targeted narrative review of the literature. A combination of structured and branching searches using several key terms (i.e., financialisation, market capitalisation, stock/equity markets, corporate governance, shareholder value/returns, common shareholder ownership, and shareholder/investor activism) were undertaken between January and May 2023 to source relevant literature. Scopus, Web of Science, and Google Scholar were used to source academic literature, and Google and company websites were used to source grey literature (including company reports and media articles). Documents found during the literature search were supplemented with the authors’ knowledge of relevant documents. Table 1 outlines the methods, metrics, and data sources used. Refer to Supplementary file 1 for a glossary of key terms used throughout this paper.

**Table 1:** Methods and data sources used in this study

Level	Methods/metrics	Description/rationale	Data sources
**Sector-level analysis**	Quantitative analysis of market capitalisation	Market capitalisation is calculated by multiplying a corporation’s share price by the number of shares it has outstanding. Market capitalisation represents the monetary value of shareholder wealth stored in a corporation in the form of corporate shares.	Compustat North America (accessed via Wharton Research Data Services)
	Quantitative analysis of the distribution of shareholder capital, focusing on total shareholder payouts in absolute terms, and total shareholder payouts relative to total revenue (i.e., shareholder value ratio)	The total monetary value of shareholder payouts was calculated by adding dividend payments and share buyback expenditure. The ‘shareholder value ratio’ is calculated by dividing the total monetary value of shareholder payouts by company revenue, a proxy for the available funds that corporations can distribute among all of its different ‘stakeholders’, including workers, shareholders, governments, and suppliers [60].	Compustat North America (accessed via Wharton Research Data Services)
	Targeted narrative review of the literature	To provide important historical context	Academic databases and grey literature materials
**Company-level analysis**	Quantitative analysis of market capitalisation	As above	Compustat North America
	Quantitative analysis of total shareholder payouts	As above	Compustat North America
	Quantitative analysis of shareholder payouts, capital expenditure, and income tax payments relative to total revenue	This type of analysis explores how much money the corporation in question distributes, relative to its total revenue, to some of its key ‘stakeholders’, including: shareholders via dividends and share buybacks; ordinary workers, with capital expenditure serving as a proxy for the long-term interests of the ordinary worker [60]; and governments in the form of income tax payments. For some governments, revenues from corporate income tax payments are important to increase their fiscal capacity to provide and fund essential infrastructure and services [125], including public health programs designed to address some of the harms caused by the UPF industry.	Compustat North America
	Targeted narrative review of annual company reports and the broader literature	To explore potential strategies or mechanisms underpinning the findings related to how the company in question has distributed its funds, as well as to provide important historical context	Publicly accessible digitalised material accessed via Internet Archive [66, 67, 126] Academic databases and grey literature materials
**Investor-level analysis**	Targeted narrative review of the literature	To explore the type and nature of shareholder campaigns taking place, and their potential influence on the governance of UPF corporations. In this paper, we focused on campaigns led or supported by so-called responsible investors and hedge fund ‘activists’.	Academic databases and grey literature materials
	Descriptive analysis of the combined share ownership of selected investors in the selected ultra-processed food corporations.	To describe the proportion of shares that particular investors hold in major UPF corporations (see below for further details on company and investor selection).	Bureau van Dijk’s Orbis database (share ownership data)
	Descriptive analysis of the number and percentage of times that selected investors vote for and against proposals targeting the selected corporations.	In this analysis, we focused on the voting behaviour of some of the world’s largest investors in terms of shareholdings (see below for further details on investor selection). One of the ways by which investors can influence the governance of corporations is through exercising their voting rights at annual shareholder meetings. In most cases, one share equals one vote, thus the voting decisions made by investors that hold the largest proportion of shares in a corporation will tend to have the greatest effect on overall shareholder support for a proposal. Specifically, we disaggregated proposals that have targeted major UPF corporations into those put forward by corporate decision-makers and those put forward by shareholders that related to an environmental, social, or governance (ESG) related issue. Within those categorised as ESG-related shareholder proposals, we identified those that related to a specific public health issue, as well as those that related to political influence and lobbying, according to the information presented in their titles.	Institutional Shareholder Services’ proxy voting dashboard U.S. Securities and Exchange Commission’s Electronic Data Gathering, Analysis, and Retrieval (EDGAR) system
	Descriptive analysis of the overall level of support for selected shareholder proposals	To describe overall investor support for all identified public health-related shareholder proposals.	Institutional Shareholder Services’ voting analytics for U.S. companies (accessed via Wharton Research Data Services)

### Study sample and sector definitions for sector-level analysis

Corporations listed on U.S. stock exchanges were included in our sample for sector-level analysis primarily because we were able to access their financial data dating back to 1950 through Compustat North America, one of the world’s largest company financial databases [53]. In comparison, we were only able to access financial data for corporations listed on stock exchanges outside of North America dating back to 1987 onwards through Compustat Global. Additionally, the financial data we extracted on corporations listed on U.S. stock exchanges were represented in U.S. dollars (USD), which enabled us to avoid complications with exchange rate conversions. Focusing on U.S. listed corporations meant that some large private food and agricultural corporations (e.g., Cargill, Mars), as well as some publicly listed food and agricultural corporations not listed on U.S. stock exchanges (e.g., Yihai International), were not included in our sample. Nevertheless, a brief scoping review revealed that our sample included a large proportion of the world’s largest food and agricultural corporations [54].

In our sector-level analysis, we opted to compare different sectors of the U.S. listed corporate food system. We organised U.S. listed corporations into the following five sectors based on their Global Industry Classification Standard (GICS) data, and in some cases their North American Industry Classification System (NAICS) data, which were extracted from Compustat North America:

i) agricultural inputs (i.e., agriculture and farm machinery, fertilisers and agricultural chemicals, agriculture-related specialty chemicals).
ii) food production, primary processing, and commodity trading (i.e., producers and traders of non-UPFs, including culinary ingredients).
iii) UPF manufacturing.
iv) food retailing (i.e., food retailers, including supermarkets and merchandise stores involved in food retail).
v) food service (i.e., restaurants and catering services).

Refer to Supplementary file 2 for an overview of NAICS and GICS, as well as how they were used in this study to inform the categorisation of corporations into the five sectors listed above. Given the aims of this study, we focused on findings pertaining to the UPF manufacturing sector and food service sector. This was because we assumed that UPFs are *central* to the revenue and profit-making models of *all* corporations active in the UPF manufacturing sector, as well as *some* corporations primarily active in the food service sector (especially fast-food corporations).

### Selection of corporations for company-level analysis

We conducted company-level analysis of a set of major corporations that, for most of their existence, have relied heavily on manufacturing and marketing UPFs to generate profit. To begin with, we identified four corporations that had consistently held top positions in terms of sector share by revenue, between 1981 and 2021, for the UPF manufacturing sector and the food service sector. We opted to select four firms and use sector share by revenue as a proxy for sector dominance because this is consistent with the top-four firm concentration ratio, a commonly used metric to measure market or sector concentration [55]. We chose the period 1981 to 2021 because we wanted to ensure that the period was sufficiently long enough to gauge long-standing sector dominance, while also ensuring that we captured the many non-U.S. based corporations that began to feature on U.S. stock exchanges from the 1980s onwards (e.g., Nestlé). Refer to Supplementary file 3 for a list of the selected corporations.

Following a preliminary scoping review of the product portfolios and corporate histories of the eight selected corporations, we considered that six out of the eight selected corporations have likely been largely dependent on manufacturing and marketing UPFs to generate profit since their inception (see Supplementary file 3). Four of these corporations, Nestlé, PepsiCo, Unilever, and Coca-Cola Co, are food manufacturing corporations. Although Unilever has sold some of its UPF operations in recent years [56], and is arguably less dependent on UPFs than the other three food manufacturing corporations, we chose to include Unilever because it has relied on UPFs to generate a large proportion of its revenues and profits for a major part of its existence [57]. McDonald’s and Yum Brands, both food service corporations, were the other two corporations selected for analysis.

### Selection of large asset management firms for investor-level analysis

To inform parts of the investor-level analysis, we chose to examine voting data pertaining to four of the world’s largest asset management firms. These were: BlackRock, which had approximately US$9.1 trillion assets under management (AUM) in March 2023; Vanguard (approximately US$7.6 trillion AUM); State Street Global Advisors (approximately US$3.6 trillion AUM), and Capital Group (approximately US$2.7 trillion AUM). These four asset management firms were chosen for two reasons. First, they were among the world’s 10 largest investors by assets under management at the time of finalising this paper [58]. Second, they were identified as holding a large proportion of shares, and thus a large amount of voting power, across the six selected corporations during a preliminary scoping analysis.

## Results

### Sector-level analysis

#### Trends in market capitalisation

**Fig. 1:**
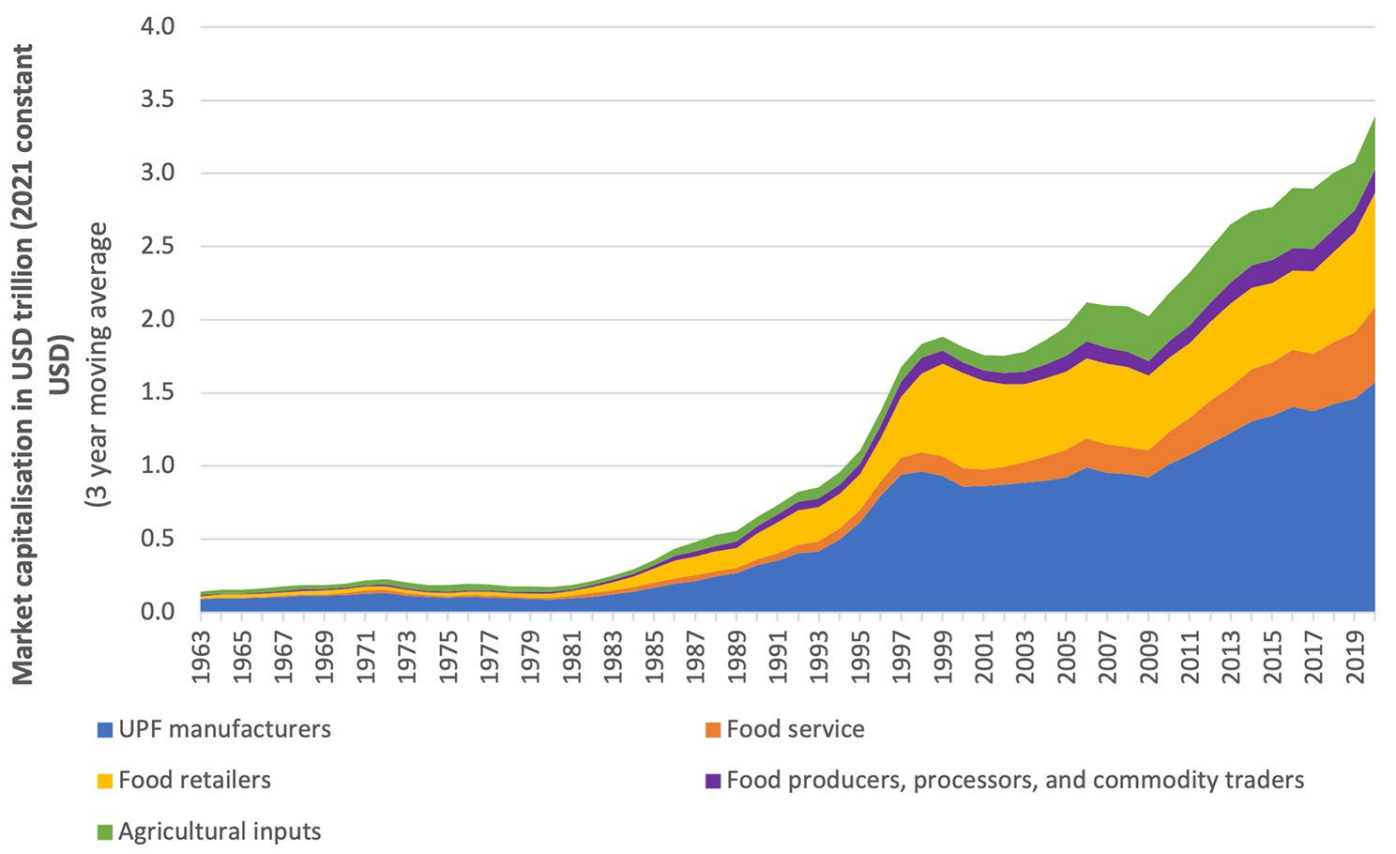
Market capitalisation trends of five major U.S. listed food sectors, 1962–2021. *Data sourced from Compustat North America, accessed via Wharton Research Data Services. Market capitalisation values = share price at end of year x common shares outstanding. Sectors defined using Global Industry Classification Standard (GICS) and North American Industry Classification System (NAICS) groupings. The agricultural input sector includes U.S. listed corporations primarily active in: agriculture and farm machinery; fertilisers and agricultural chemicals; and specialty chemicals if identified as being linked with agriculture. The food production, primary processing, and commodity trading sector includes U.S. listed corporations primarily active in industries related to the production and trade of non-ultra-processed-foods, including culinary ingredients. The UPF manufacturing sector includes U.S. listed corporations primarily active in food industries that mostly produce and market UPFs. The food retailing sector included U.S. listed corporations primarily active in food retail, including supermarkets, as well as hypermarkets and merchandise stores if identified as being involved in food retail. The food service sector includes corporations active in the restaurant and catering services industries

Between 1962 and 2021, the aggregate market capitalisation of agri-food corporations in the five sectors increased more than 30-fold in real terms, from around US$120 billion (2021 USD) to US$3.7 trillion (Fig. 1). Over this period, the market capitalisation of the UPF manufacturing sector – by far the largest of the five sectors in terms of market capitalisation – was seen to increase from around US$80 billion to more than US$1.6 trillion in real terms. In comparison, the relative contribution of the UPF manufacturing sector to the aggregate market capitalisation declined from approximately 68% in 1962 to 44% in 2008, thereafter remaining relatively stable. The market capitalisation of the food service sector, which is partly comprised of fast-food restaurants that are dependent on UPFs to generate sales, increased from approximately US$0.8 billion in 1962 in real terms (approximately 1% of aggregrate market capitalisation) to nearly US$580 billion in 2021 (approximately 16% of aggregrate market capitalisation).

The combined market capitalisation of the five sectors began to surge in the 1980s, in part because the share prices of many major agri-food corporations increased considerably during this period. For example, Coca-Cola Co’s share price increased seven-fold during the 1980s [59]. Another potentially important factor was that, during the 1980 and 1990 s, many non-U.S. based agri-food corporations, including Nestlé, began to feature on U.S. stock exchanges.

#### Trends in the distribution of shareholder capital

**Fig. 2:**
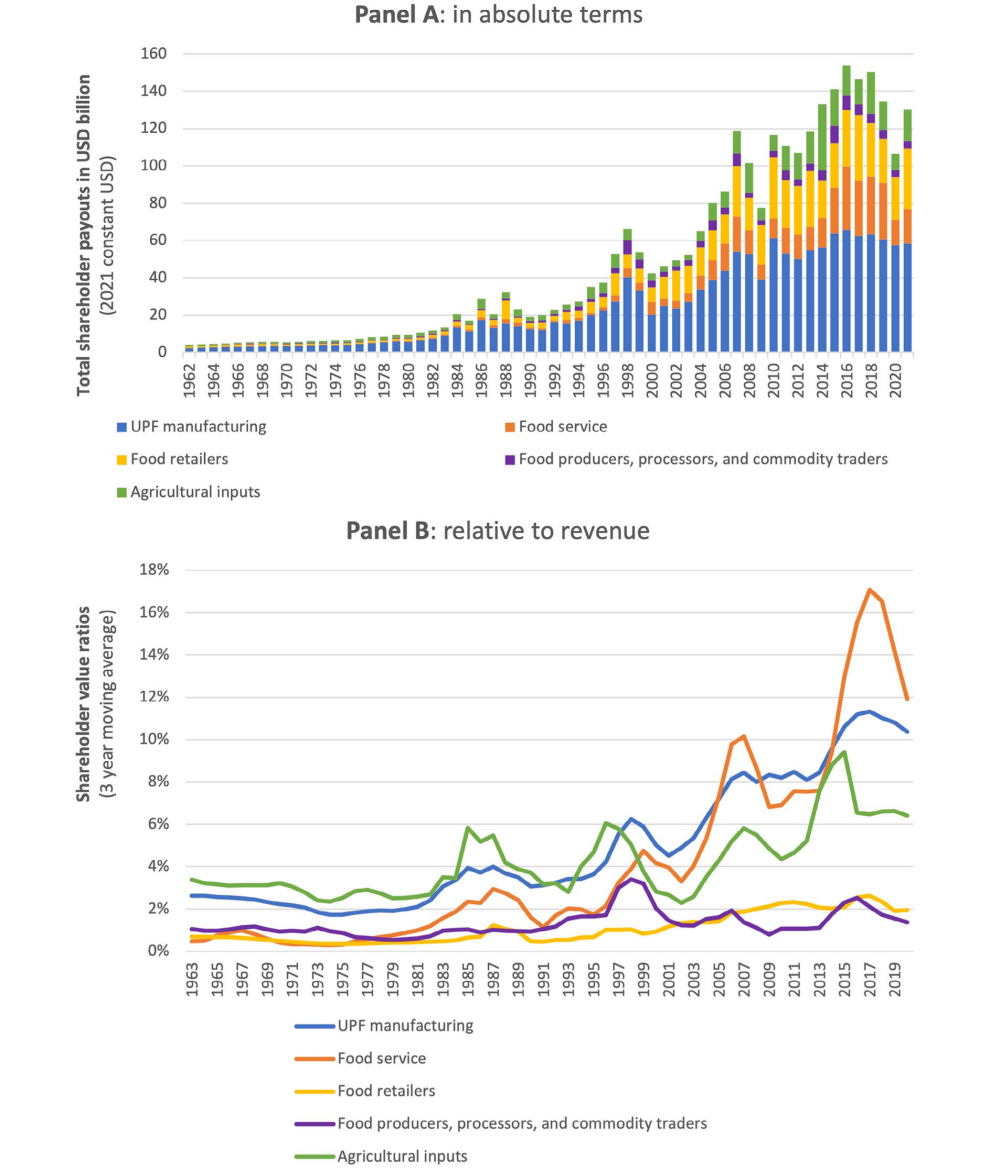
Shareholder payouts made by five U.S.-listed food and agricultural sectors in absolute terms (Panel A) and relative to total revenue (Panel B), 1962–2021. *Data sourced from Compustat North America, accessed via Wharton Research Data Services. Shareholder value ratios = (dividends paid + value of share repurchases)/ total revenue. Share repurchase data from Compustat may include data on purchase of preferred stock. Sectors defined using Global Industry Classification Standard (GICS) and North American Industry Classification System (NAICS) groupings. The agricultural input sector includes U.S. listed corporations primarily active in: agriculture and farm machinery; fertilisers and agricultural chemicals; and specialty chemicals if identified as being linked with agriculture. The food production, primary processing, and commodity trading sector includes U.S. listed corporations primarily active in industries related to the production and trade of non-ultra-processed-foods, including culinary ingredients. The UPF manufacturing sector includes U.S. listed corporations primarily active in food industries that mostly produce and market UPFs. The food retailing sector included U.S. listed corporations primarily active in food retail, including supermarkets, as well as hypermarkets and merchandise stores if identified as being involved in food retail. The food service sector includes corporations active in the restaurant and catering services industries

Of the approximately US$2.9 trillion (2021 USD) of shareholder capital distributed by the five sectors between 1962 and 2021, more than 50% (US$1.5 trillion in 2021 USD) was distributed by the UPF manufacturing sector, while approximately 13% (US$0.4 trillion in 2021 USD) was distributed by the food service sector. In 2021, U.S. listed corporations active in the five selected sectors distributed approximately US$130 billion in shareholder capital (via dividends and share buybacks) (Fig. 2, panel A). UPF manufacturing corporations and food service corporations were responsible for nearly 45% (US$58 billion) and 14% (US$19 billion) of this monetary value, respectively. Excepting several financial crises, including the global financial crisis (2007–2009) and that related to the COVID pandemic (2020), the value of shareholder capital distributed by these five sectors generally trended upwards over the 60-year period of analysis.

Since the early 1990s, the UPF manufacturing sector and the food service sector have, in general, distributed a greater proportion of their revenue to their shareholders compared to the other three agri-food sectors (Fig. 2, Panel B). Drawing from the work of others [60], we refer to this ratio as the ‘shareholder value ratio’ because it serves as a useful proxy for the percentage of funds that corporations distribute to their shareholders relative to the amount they distribute among their other ‘stakeholders’, including workers [60]. During the three-year period between 2019 and 2021, the UPF manufacturing sector and the food service sector distributed the equivalent of 10.4% and 11.9% of their revenue to their shareholders, compared to 3.1% and 1.1% during the three-year period between 1990 and 1992. Between 2019 and 2021, the agricultural input sector, the food production, processing, and commodity trading sector, and the food retailing sector distributed the equivalent of 6.4%, 1.4%, and 2.0% of their revenue to their shareholders.

### Company-level analysis

#### Trends in market capitalisation

Today’s largest UPF manufacturing corporations by market capitalisation were among the first of all existing food and agricultural corporations to be publicly traded. Nestlé, the frontrunner in this respect, was first publicly traded on the Zurich Stock Exchange in 1873 [61]. 46 years later, Coca-Cola Co and PepsiCo both made their first public offerings on the New York Stock Exchange (NYSE) in 1919 [62, 63]. In 1930, newly formed Unilever, created when Dutch company Margarine Unie merged with British company Lever Brother, was listed on the Amsterdam stock exchange [64]. It was not until after the Second World War when today’s largest major UPF food service corporations began to emerge. In 1965, McDonald’s made its first public offering on the NYSE [65]. PepsiCo acquired Pizza Hut, Taco Bell, and Kentucky Fried Chicken in 1977, 1978, and 1986, respectively, before spinning off its fast food restaurant operations in 1997 under the publicly listed corporation Tricon Global Restaurants [66]. After merging with U.S. company Yorkshire in 2002, Tricon Global Restaurants changed its name to Yum Brands [66].

**Fig. 3:**
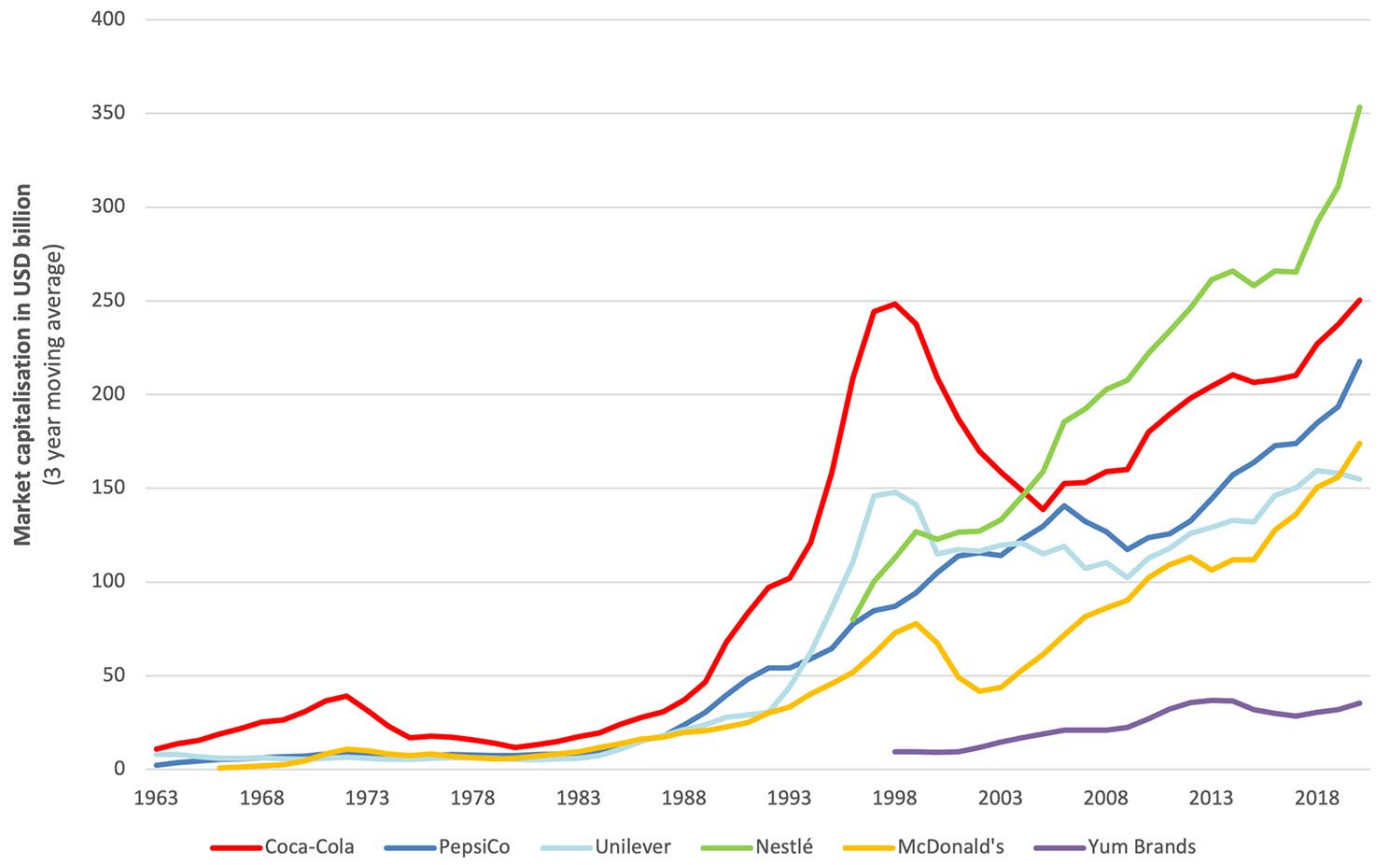
Market capitalisation trends of six major UPF corporations, 1962–2021. *Data sourced from Compustat North America, accessed via Wharton Research Data Services. Market capitalisation values = share price at end of year x common shares outstanding

Figure 3 shows the trends in market capitalisation of the six abovementioned major UPF corporations. Beginning in the early 1980s, the market capitalisation of Coca-Cola Co, and to a lesser extent PepsiCo, Nestlé, Unilever, and McDonald’s, began to surge. By the end of 1997, Coca-Cola Co’s market capitalisation reached approximately US$260 billion (in 2021 USD), placing the company in the world’s top-five largest corporations by market capitalisation at that time [67]. Around the turn of the 20th century, however, the market capitalisation values of Coca-Cola Co, and to a lesser extent Unilever and McDonald’s, decreased considerably. Among other factors, some commentators attributed the fall of Coca-Cola Co’s market capitalisation to issues such as a reorganisation of the company’s management structure, a probe led by the U.S. Securities and Exchange Commission into its accounting practices, and the declining sales of its flagship product, Coca Cola [68]. From the mid-2000s till 2021, the market capitalisation of Nestlé, PepsiCo, Coca-Cola Co, Unilever, and McDonald’s were seen to increase along a relatively similar trajectory. The combined market capitalisation of Nestlé, Coca-Cola Co, PepsiCo, McDonald’s, Unilever, and Yum Brands reached nearly US$1.3 trillion at the end of 2021, representing more than 34% of the combined market capitalisation of the five agri-food sectors analysed.

#### Trends in the distribution of shareholder capital

In 2021, Nestlé, Coca-Cola Co, PepsiCo, McDonald’s, Unilever, and Yum Brands distributed a combined total of approximately US$45 billion to their shareholders via dividends and share buybacks. This amount represented approximately 34% of the aggregrate shareholder payouts made by all five sectors analysed.

**Fig. 4:**
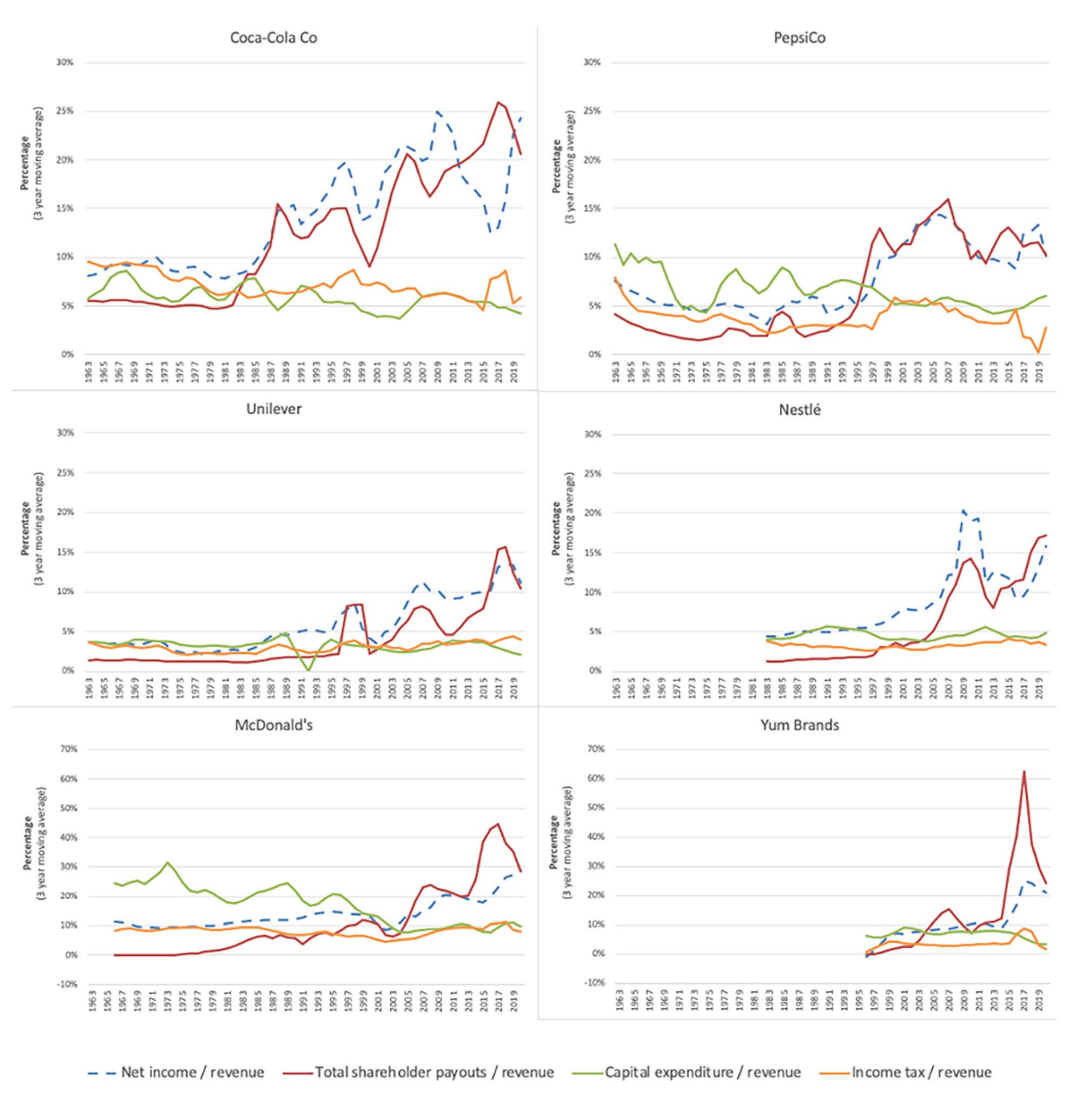
Net income, shareholder payouts, capital expenditure, and income tax paid by six major UPF-dependent corporations relative to their revenue, 1962–2021. Data sourced from Compustat North America, accessed via Wharton Research Data Services. Total shareholder payouts = dividends paid + value of share repurchases. Share repurchase data from Compustat may include data on purchase of preferred stock. N.b. the y-axes vary for McDonald’s and Yum Brands compared to the other corporations

At different points in time, the six major UPF corporations analysed in this study began to transfer greater amounts of money to their shareholders relative to their total revenue, capital expenditure (a proxy for the long-term interests of the ordinary worker), and income tax payments (Fig. 4). Coca-Cola Co was the first of these six corporations to reach this inflection point, which occurred in the early 1980s. This happened around the same time that the then-Chief Executive Officer (CEO) of Coca-Cola Co Roberto Goizueta reported that the company’s ‘*primary objective will continue to be the maximization of shareholder value*’ [67]. While this sort of rhetoric was becoming increasingly commonplace in the 1980s, at least in the U.S. [69], it nevertheless represented a distinct departure from the rhetoric of the company’s earlier CEOs. In 1959, for instance, William Robinson contended that it was an error for executives to put shareholders ‘*first, last, and all the time*’, and that corporations needed to also serve workers, customers, and communities [70].

The six major UPF corporations operationalised the objective of maximising shareholder returns in the *short-term* in several ways, albeit to varying degrees. First, at different points in time, their annual dividend payments began to surge (Fig. 4). Relative to 1982, the annual dividend payments made in 2021 by Coca-Cola Co (US$7.3 billion), PepsiCo (US$5.9 billion), Nestlé (US$8.4 billion), Unilever (US$5.1 billion) and McDonald’s (US$3.9 billion) increased by more than 9 times, 16 times, 21 times, 14 times, and 34 times, respectively, in real terms. In 1999, a year after selling part of its chemical operations, Unilever also paid a ‘special dividend’ of 5 billion GBP, which, at the time, was the world’s largest ever single payment to shareholders [71]. In 2021, Yum Brands annual dividend payments reached US$0.6 billion, nearly 5 times larger than when it first began paying dividends in 2004.

**Fig. 5:**
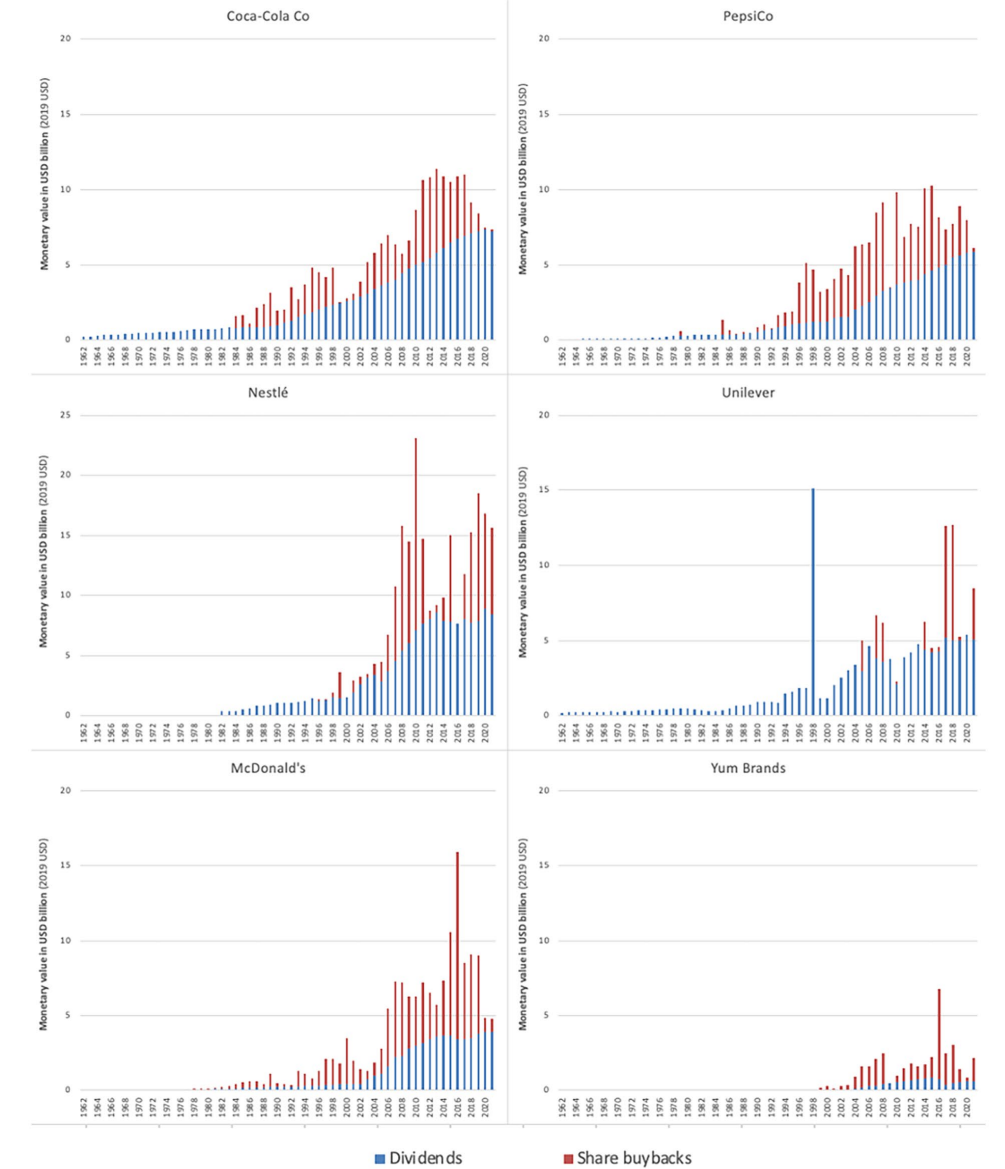
Monetary value of dividends and share buybacks made by six major UPF-dependent corporations, 1962–2021. Data sourced from Compustat North America, accessed via Wharton Research Data Services. Share repurchase data from Compustat may include data on purchase of preferred stock

Second, like many publicly listed corporations in diverse sectors, the corporations began to undertake large share buyback programs (Fig. 5). As with dividends, share buybacks –when a corporation buys back its own shares on the open stock market – is a practice that transfers money from the ‘real economy’ to corporate shareholders [32]. Share buybacks also influence financial metrics commonly linked to executive remuneration [32]. Shortly after the practice effectively became legalised in the U.S. in 1982 [72], Coca-Cola Co began its first of many large share buyback programs. By 1990, Coca-Cola Co had already spent approximately US$8 billion on share buybacks in real terms (2021 USD). After beginning its first formal share buyback program in 2005 [73], Nestlé, the UPF corporation that has spent the most on share buybacks in the last decade, bought back nearly US$100 billion worth of its own shares between 2006 and 2021 in real terms.

Third, some of the major UPF corporations underwent major restructuring to boost their net profit margins (net income relative to revenue), a ratio that has tracked closely to their ‘shareholder value ratios’ (Fig. 5). In 1999, for instance, Coca-Cola Co underwent a restructuring ostensibly to reduce production costs, with more than 5,000 jobs cut from the company’s global workforce [70]. As another example, in 2020, Unilever underwent a major restructure as part of its so-called ‘Path to Growth’ strategy [74]. Within ten years of the strategy’s implementation, the company had cut more than 90,000 jobs. In response to falling profits and revenues in the early 2010s, McDonald’s announced a ‘turnaround plan’, involving the ‘stripping’ away of layers of management, as well as the ‘refranchising’ of its restaurants (i.e., selling company-owned restaurants to franchisees) to increase profitability [75, 76]. Similarly, in 2016, Yum Brands decided to spin off its Chinese operations into a separate publicly listed company, in part to facilitate the company’s refranchising strategy [77]. Before doing so, the company sought to ‘return cash to shareholders’ by buying back US$6.1 billion worth of its own shares, a move made possible through the issuance of large amounts of debt [78].

Fourth, as with many major transnational corporations, the major UPF corporations’ profit margins also likely benefited from declining corporate income tax rates around the world, especially up until the 2010s [79]. Evidence also suggests that the major UPF corporations have actively sought to minimise their income tax obligations, including by shifting their profits from high-tax jurisdictions to lower-tax jurisdictions [80–83]. While Fig. 5 does not show the effective tax rates paid by the major UPF corporations, it does show that, relative to total revenue, their income tax payments, which make up a large proportion of the money that these corporations transfer to governments, have fallen and/or remained relatively low.

### Investor-level analysis

#### The influence of investors on the governance of major UPF corporations: responsible investors

In our analysis of voting data, we identified 14 separately filed public health-related shareholder proposals that were put to a vote at the annual shareholder meetings of the selected UPF corporations between 2012 and 2022. All 14 targeted Coca-Cola Co, PepsiCo, and McDonald’s. In some cases, similar proposals were filed with multiple corporations and during different years. For example, Harrington Investments filed seven separate shareholder proposals calling on the corporation’s decision-makers to commission an independent review of the sugar-related health impacts of its products – three times with Coca-Cola Co (2019, 2020, 2021), twice with PepsiCo (2020, 2021), and twice with McDonald’s (2020, 2021). All 14 public-health related proposals received insufficient support to be passed. We found that the average support for the identified public health-related proposals was only 10.5%. The highest level of support we found was 13.5%, which was for a proposal filed with PepsiCo in 2022 entitled ‘Report on Public Health Costs of Food and Beverage Products’.

We identified that some responsible investors have also attempted to influence the governance of major UPF corporations through direct engagement with corporations, such as face-to-face meetings, videoconferences, telephone calls, and written communication, often as part of a coalition. As an example, fund managers from Switzerland-based Pictet Asset Management have directly engaged with major UPF manufacturers such as Nestlé, reportedly to increase the share of ‘healthy products’ within their portfolios [84]. Similarly, fund managers from UBS Asset Management reportedly led a collaborative engagement involving 30 investors with Chinese food corporation China Mengniu to discuss, among other things, issues relating to nutrition [85]. As with some other investors, Pictet and UBS Asset Management receive technical guidance from the not-for-profit organisation Access to Nutrition Initiative (ATNI), which claims to assess the nutritional quality of a corporation’s portfolio by analysing the extent to which it is comprised of fruit and vegetables, as well as the levels of fat, salt, sugar and other components within individual products [86].

Given that most investors do not disclose the full details of their engagement with corporations, it is difficult to assess the extent to which responsible investors have influenced the governance of major UPF corporations to promote public health and nutrition. In 2022, though, the non-governmental organisation ShareAction announced that an investor coalition it had led had managed to pressure Unilever into committing to publicly report on the healthfulness of the corporation’s food products against a range of government-endorsed nutrient-profiling models [87].

#### The influence of investors on the governance of major UPF corporations: hedge funds

We found that all six major UPF corporations have been targeted by so-called hedge fund ‘activists’. Hedge fund ‘activists’ have managed to considerably influence the governance of major UPF corporations, at least in recent years. In 2016, for instance, hedge fund Corvex management bought a stake in Yum Brands, and then afterwards managed to secure a board position for its CEO Keith Meister [88]. Before long, Keith Meister disclosed plans for the company to spin off its Chinese operations which, as discussed in the previous section, were later enacted [88]. The rationale behind this corporate restructure was that it would provide shareholders with a steady stream of income from royalties generated through the ‘refranchising’ of its company-owned restaurants in China [88]. As another example, in 2017, hedge fund Third Point bought a US$3 billion stake in Nestlé. The hedge fund soon began to apply considerable pressure on Nestlé’s board and management in an attempt to increase the company’s profitability, share price, and shareholder returns [89, 90]. Within a short period of time, Nestlé had reportedly carried out many of Third Point’s demands, including the implementation of a large share buyback program [91]. In a similar fashion, hedge fund company Trian Fund Management chose to ‘exit’ PepsiCo after the company’s share price and dividend payments had surged, with the hedge fund claiming that it played a key role in driving these shareholder gains [92]. The same hedge fund opted to target Unilever in 2022, acquiring a 1.5% stake in the company. Shortly afterwards, and amid rising shareholder discontent regarding the company’s lagging share price, Trian Fund Management co-founder Nelson Peltz was given a position on Unilever’s board [93].

#### Share ownership and voting behaviour of four of the world’s largest asset management firms

**Fig. 6:**
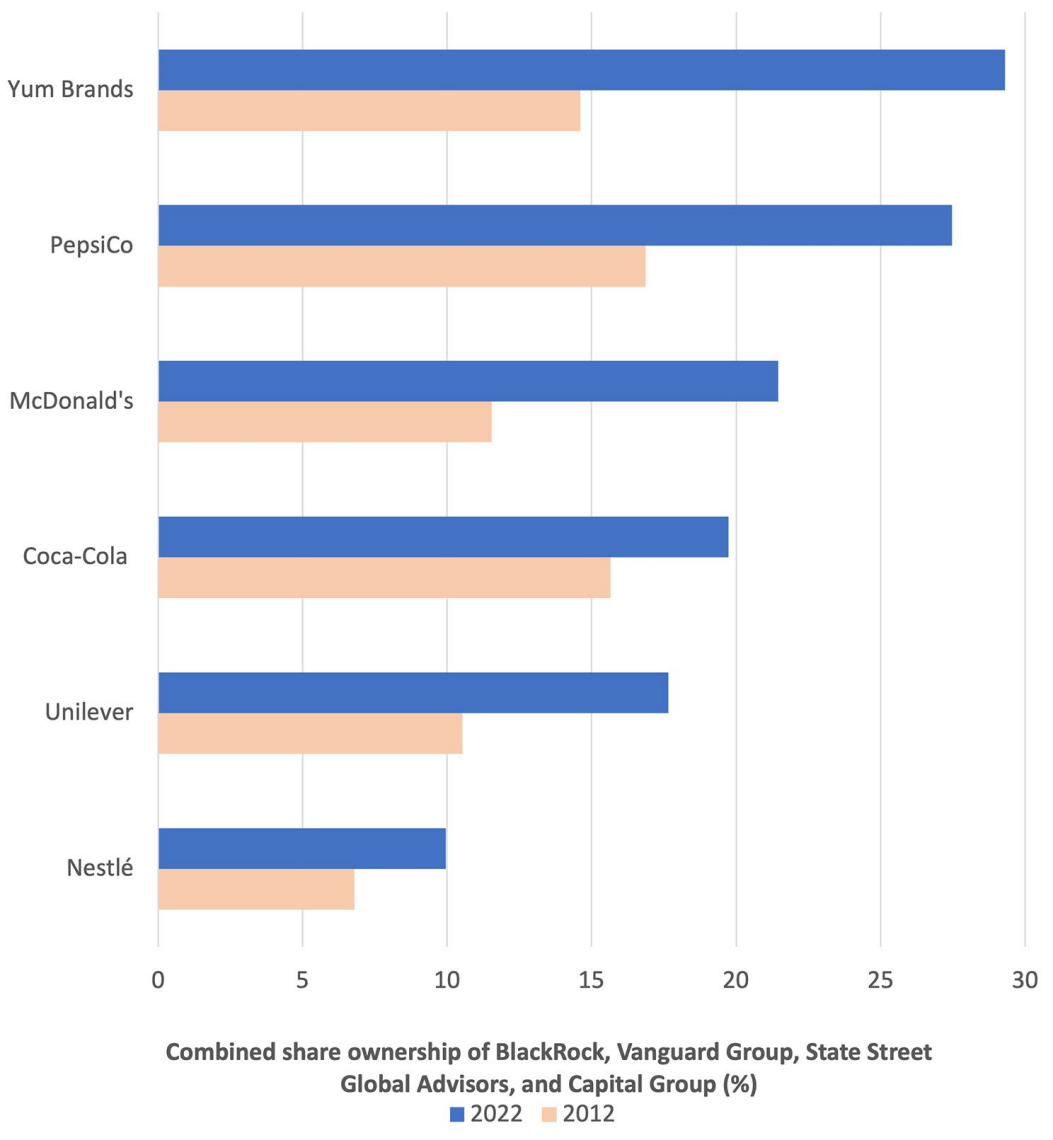
Combined share ownership of four of the world’s largest asset managers in six major ultra-processed food corporations, 2012 vs. 2022. Data sourced from Bureau van Dijk’s Orbis database

Figure 6 demonstrates that the voting behaviour of BlackRock, Vanguard, State Street, and Capital Group likely has a large influence on overall shareholder support for proposals targeting the six major UPF corporations. In combination, these four investors held approximately 29.3% of shares in Yum Brands in 2022 (compared to 14.6% in 2012), 27.5% of shares in PepsiCo (16.9% in 2012), 21.5% of shares in McDonald’s in 2022 (11.5% in 2012), 19.7% of shares in Coca-Cola Co (15.7% in 2012), 17.7% of shares in Unilever (10.5% in 2012), and 10.0% of shares in Nestlé (6.8% in 2012).

Table 2 shows the number and percentage of times that BlackRock, Vanguard, State Street and Capital Group voted for and against proposals put to a vote by the board of directors, as well as the public health and other environment, social, and governance (ESG) related proposals put to a vote by shareholders, during annual shareholders between 2012 and 2022. All four asset management firms were found to vote overwhelmingly in favour of the proposals put forward by the board of directors, most of which related to remuneration and board elections. We also found that the four investors did not vote against any of the four proposals put forward by the board of directors of the two Western Europe-based corporations – Nestlé and Unilever – relating to increasing shareholder payouts and approving political donations and lobbying expenditure.

**Table 2:** Number and percentage of times that five of the world’s largest institutional investors voted for and against proposals at annual shareholder meetings, 2012–2022

	Proposal put forward by the board of directors		Shareholder proposals on a specific public health issue		Shareholder proposals on lobbying and political influence		All ESG-related shareholder proposals	
	For	Against	For	Against	For	Against	For	Against
BlackRock	1151 (98.8%)	14 (1.2%)	0 (0%)	14 (100%)	0 (0%)	10 (100%)	1 (1.1%)	89 (98.9%)
Vanguard	1164 (99.7%)	4 (0.3%)	0 (0%)	12 (100%)	0 (0%)	7 (100%)	0 (0%)	77 (100%)
State Street	1113 (97.4%)	30 (2.6%)	0 (0%)	12 (100%)	0 (0%)	10 (100%)	3 (3.4%)	84 (96.6%)
Capital Group	1058% (97.2%)	31 (2.8%)	0 (0%)	13 (100%)	0 (0%)	10 (100%)	20 (23.8%)	64 (76.2%)

Data sourced from filings made to the U.S. Securities and Exchange Commission. Data includes ‘for’ or ‘against’ votes, but excludes abstentions, votes withheld, ‘take no action’ votes, and votes on ‘transact other business’ proposals. In some cases, vote data were unable to be found. The six major UPF corporations included in this analysis were: Nestlé, PepsiCo, Unilever, Coca-Cola Co, McDonald’s, and Yum Brands

Excepting abstentions and votes withheld, these four investors were found to vote against all identified public health-related shareholder proposals. Similarly, they were also found to vote against all identified shareholder proposals relating to political contributions and lobbying, with the majority of these proposals calling for the commissioning of a report on the corporation’s current political activities in order to increase transparency. More broadly, we found that BlackRock, Vanguard, and State Street voted in favour of the identified ESG-related proposals 1.1%, 0%, and 3.4% of the time, respectively (excepting abstentions and votes withheld). Capital Group was found to vote in favour of 23.8% of the identified ESG proposals, with the majority of these (12/20) relating to increasing the rights of shareholders (e.g., reducing the threshold for shareholders to call a special meeting).

## Discussion

### Overview

This study showed that a substantial proportion of the shareholder wealth and income derived from the U.S. corporate food system has been and continues to be accumulated in and generated by the UPF manufacturing sector. Furthermore, since the 1980s, the UPF manufacturing sector and the food service sector, which is partly comprised of fast-food restaurants dependent on UPFs to generate sales, have been transferring an increasing amount of money towards their shareholders relative to total revenue. While this trend was seen across most of the agri-food sectors analysed, we found that the proportion (i.e., the ‘shareholder value ratio’) was substantially higher in these two sectors. In particular, the study strongly suggests that the decision-makers of six major UPF corporations – Nestlé, PepsiCo, Unilever, Coca-Cola Co, McDonald’s, and Yum Brands – have been increasingly operationalising the objective of maximising shareholder returns in the *short-term*. We identified three major ways by which this has happened: (i) by increasing annual dividend payments; (ii) by adopting large-scale share buyback programs; and (iii) by restructuring the company to reduce production costs (including by cutting jobs), along with maintaining relatively low levels of capital expenditure. It also appears that these corporations were able to lower or keep their income tax obligations relatively low, at least until the 2010s.

The study also highlighted that different types of investors with varied perspectives on how best to maximise shareholder value are seeking to influence the governance of major UPF corporations. Specifically, we found that hedge fund managers have been particularly effective at influencing the governance of these corporations to maximise their returns in the *short-term*. In comparison, self-declared responsible investors seeking to improve population diets have had only limited success, not least because such investors have generally been unable to gain the support of some of the world’s largest asset management firms, at least with respect to shareholder proposals.

The findings of this study have several important implications for public health advocates, researchers, and policy-makers. Importantly, the operationalisation of ‘shareholder primacy’ by major UPF corporations undermines the claims made by these corporations that they are contributing to sustainable development, such as by ‘creating shared value’ or building sustainable economies [38–40]. It is indeed very difficult to reconcile such claims with the ways in which the same corporations are transferring an increasing proportion of the money they generate through UPF sales, a large proportion of which comes from the income of lower-income households in high-income countries and citizens in low-and-middle countries, to shareholders and the ultimate owners of assets under management, a group over-represented by the wealthy in high-income countries [94–97]. This pattern of ‘maldistribution’ reinforces, and is reinforced by, the ways in which the social and ecological harms driven by major UPF corporations disproportionately burdens disadvantaged population groups and low and middle-income governments [2, 98]. The study thus further delegitimises the involvement of major UPF corporations in governance arrangements that relate to addressing the social and environmental impacts of contemporary population diets, and reinforces the need for robust conflict of interest mechanisms that target the involvement of major UPF corporations in such arrangements, such as those recently implemented by the United Nations Children’s Fund [99].

The study also highlights some of the potential limitations of initiatives involving responsible investors seeking to improve population diets and public health. Regarding public health activism in the form of shareholder proposals, it is worth noting that the legal requirements that shareholders must meet in order to able to file a proposal vary considerably among jurisdictions [100]. For instance, in countries such as France and the Netherlands, the use of shareholder proposals to prompt changes in corporate behaviour is heavily restricted by law [100]. In comparison, the legal requirements that need to be met to file a shareholder proposal in the U.S. are relatively less restrictive, which perhaps partly explains why all the public health-related shareholders proposals that we identified targeted U.S. based UPF corporations. In any case, when such proposals requesting direct action from the decision-makers of major UPF corporations have been successfully filed, they have invariably failed to gain majority support. As our analysis indicated, an important reason for this is that large asset management firms like BlackRock and Vanguard with considerable voting power mostly vote against such proposals.

Besides shareholder proposals, responsible investors can influence corporate governance in other ways, such as by directly engaging with corporations. However, most responsible investors do not disclose the full details of their engagement with corporations. Thus, it is difficult to assess the effectiveness of this type of investor engagement, as well as the extent to which it aligns with best-available evidence. Moreover, based on the details of a few cases of investor engagement that have been made publicly accessible, concerns have been raised about the possibility that such engagement might be unintentionally impeding broader efforts to improve population diets. For example, some claim that the various nutrient profiling models used to inform some investor initiatives could be causing harm, such as by conferring many UPF products with a so-called ‘health halo’ and thus inadvertently promoting their consumption over healthier alternatives [28].

More broadly, the study indicates that investor initiatives genuinely seeking to improve population diets and public health must contend with powerful investors, especially hedge fund managers, seeking to directly influence corporate governance to maximise their returns in the *short-term.* This is not to say that the interests of hedge fund ‘activists’ and responsible investors are always in tension. However, a clash between these investor groups may arise in cases where investors seeking to maximise *long-term* shareholder value push for incremental measures that have the potential to jeopardise the corporation’s capacity to maximise *short-term* shareholder value. Problematically, at least from a public health perspective, the overwhelming evidence suggests that, when such cases arise, *short-termism* almost always wins [46]. The same dilemma also faces the senior decision-makers of UPF corporations themselves seeking to implement social and environmental initiatives, as illustrated by the recent dismissal of Emmanuel Faber as CEO of Danone. In 2021, Faber, was terminated by the company’s board as a result of a hedge fund-led campaign, which contended that Faber’s focus on sustainability was jeopardising the company’s *short-term* financial performance [101].

### Policy recommendations

In this section, we highlight four synergistic strategies that can help to address the global rise of UPF-dominant diets through protecting population diets from the extractive forces of financialisation.

First, we argue that measures that shift the real or perceived purpose of the corporations that produce UPFs away from maximising shareholder value warrant serious consideration. Despite the emergence of new voluntary models of corporate purpose that supposedly encourage the creation of ‘stakeholder value’ and ‘profits with purpose’ [102–105], there is no evidence that indicates that such models will be sufficient to improve population diets [19]. Instead, some scholars have called for governments to redefine corporate purpose under law in order to make it *obligatory* for corporate directors to fully consider the interests of a broader range of actors in their decision-making [36, 105]. Among other benefits, this law would likely provide a legal framework to counter the powerful norm of ‘shareholder primacy’ that pervades corporate governance today. However, it remains unclear exactly how the corporate decision-makers of UPF corporations would be able to reconcile their pursuit of profits with the interests of citizen-consumers and the general public, given that high dietary exposure to UPFs is associated with substantial health and environmental harms. Indeed, it might be the case that many UPF corporations, and corporations active in health-harming industries more broadly, cannot be appropriately repurposed to ‘solve the problems of people and planet profitably’ [106]. As such, more radical measures might need to be considered, such as bringing major UPF corporations under public ownership with the view of either winding them down, or at least governing them in a way that better aligns with the public interest [107].

While UPF corporations continue to pursue the twin goals of maximising profits and shareholder returns, exploitative corporate practices and government policies that allow corporations to produce UPFs with high profit margins, but still often at a consumer price lower than healthier alternatives, warrant attention. Intervention strategies that disincentivise the harmful practices that are used to maximise profits in the short-term are likely to encompass robust legislative actions and enforcement mechanisms. In this respect, illustrative examples include bans on the predatory marketing of unhealthy foods to disadvantaged populations [108], scaling-up and strengthening national government regulations in accordance with the International Code of Marketing of Breastmilk Substitutes [109], and clear and robust food labelling and consumer laws that increase the accuracy and appropriateness of information provided to citizen-consumers about UPF products [110, 111]. Similarly, measures that force UPF corporations to *internalise* the costs they externalise onto society appear to be well justified. Taxes on sugary drinks provide an illustrative example of such a measure [112], and the application of a similar style of tax to a broader range of unhealthy foods warrants further consideration [113]. More broadly, cross-sectoral actions, such as transitioning agricultural policies away from providing major UPF corporations with heavily subsidised inputs [114], as well as strengthening and rethinking competition law and enforcement (e.g., by treating unsustainable corporate practices as abuses of market dominance in cases where it provides an unfair competitive advantage), deserve exploration [115].

Third, governments, civil society, and business actors should support alternative food economies to help counter the dominance of corporate food systems that, over the course of many decades, have become heavily structured and incentivised to produce and market UPFs [22, 116]. A key component of this strategy could entail increasing the role and contribution of non-corporate food businesses, such as producer co-operatives and social enterprises, in the production and distribution of diverse, sustainable foods. Governments have an important role to play in supporting alternative food businesses and economies, including by developing and scaling-up supportive infrastructure (e.g., produce markets that encourage farmer participation), as well as by implementing legislation to support the development and scaling-up of co-operatives and social enterprises [116].

Fourth, governments and investors should support and promote sustainable finance initiatives that counter ‘shareholder primacy’ and support alternative food economies. As an example, this strategy could entail much stricter corporate reporting rules, such as those outlined by the European Commission’s Corporate Sustainability Reporting Directive, which broaden the scope of what corporations must disclose with respect to the implementation of their environmental, social, and governance policies [117]. Among other potential benefits, this measure could provide responsible investors with the necessary information to ensure that their investments align with their mandates and values. At the same time, governments should also strictly regulate the ESG claims made by financial actors about their financial products and investment strategies.

Fundamentally, alternative food economies will need to be sufficiently funded so that they can achieve their social and environmental objectives, as well as funded in a way so that they are protected from extractive financial actors seeking to maximise their returns. In this respect, large-scale public investment in alternative food economies will likely be crucial. Importantly, this type of public investment can provide governments with enormous benefits, not least by contributing to the achievement of multiple policy objectives (e.g., by increasing jobs, reaching climate change targets, reducing government expenditure on healthcare costs) [118]. Where necessary, social finance initiatives, such as community finance loans, that support alternative food economies could serve as an important complement to public investment [119, 120].

### Strengths, limitations, and research opportunities

A strength of this study is that it provided a novel approach to analysing trends in the corporate governance of UPF corporations. The analysis used a large and diverse range of data from various sources, including corporate financial databases and company reports, that are rarely integrated in public health research.

The study has several important limitations. First, we only analysed corporations listed on U.S. stock exchanges. While U.S. listed corporations account for the majority of global corporate equity in terms of monetary value, the examination of the behaviour and governance of corporations listed on stock exchanges outside of the U.S., as well as private corporations such as Mars and Cargill, would likely provide deeper insight on the subject.

Second, beyond analysing voting data, we did not explore in detail other mechanisms by which large investors influence the governance of major UPF corporations. Future work could seek to address this gap, such as by critically examining the ways in which direct shareholder engagements and ESG capital allocation strategies shape the behaviour of UPF corporations.

Third, a limitation of this paper is that it did not analyse *why* major UPF manufacturing and fast-food corporations tend to have very high market capitalisations, as well as pay out a larger proportion of their revenues to their shareholders, relative to other food and agricultural sectors. This is an important research gap that needs addressing. We argue that one hypothesis worth testing is that these corporations have become very effective at generating ‘rents’, referring to the generation of financial returns based on the ownership of a scarce asset (e.g., brands, real estate upon which fast food franchisees are operated) [121]. These considerable rent-generating capacities have likely been driven by, as well as reinforced by, powerful financial actors seeking to translate these ‘rents’ into shareholder returns [122]. Yet, as suggested by the recent divestments and spinoffs of UPF operations by several large corporations [123, 124], the pressure placed on UPF corporations by extractive financial forces to increasingly generate rents and shareholder returns could be exposing ‘cracks’ in such capacities. The extent to which these ‘cracks’ could be leveraged by public health advocates also warrants exploration.

Fourth, it was not possible to account for the fact that many agri-food corporations are active in multiple sectors, and those manufacturing UPFs are co-dependent on a ‘corporate ecosystem’ of other food systems sectors and industries. We recognise, for instance, that many UPF manufacturers also have food production and primary processing operations, and vice versa. Likewise, many food retailers have their own home-brand products, many of which are UPFs, often made by manufacturers under contract. Thus, it should be noted that the sector classifications in our study pertain to the primary operations of the corporations in question, rather than their entire operations. Relatedly, we also recognise that major corporations in all food and agricultural sectors likely profit from UPFs, albeit in different ways and to various degrees. In this regard, future work could examine the extent to which major corporations in various sectors (e.g., agricultural inputs, commodity trading, and food retail) profit from the production and consumption of UPFs.

## Conclusion

The operationalisation of ‘shareholder primacy’ by major UPF corporations has driven inequity and undermines their claims that they are creating ‘value’ for diverse actors. Investors actively seeking to maximise their returns in the *short-term*, especially hedge fund ‘activists’, have been influential in reinforcing this form of corporate governance. Fundamentally, our study highlights the need for government and collective actions that protect population diets and food systems from the extractive forces of financialisation as part of efforts to address the global rise of UPFs in human diets.

### Electronic supplementary material

Below is the link to the electronic supplementary material.

Supplementary Material 1

Supplementary Material 2

Supplementary Material 3

## Data Availability

The data that support the findings of this study are available from various third parties but restrictions apply to the availability of these data, which were used under license for the current study, and so are not publicly available.

## Abbreviations

ATNI: Access to Nutrition Initiative
AUM: Assets under management
CEO: Chief Executive Officer
EDGAR: Electronic Data Gathering, Analysis, and Retrieval
ESG: Environmental, social and governance
GICS: Global Industry Classification Standard
NAICS: North American Industry Classification System
NYSE: New York Stock Exchange
UPFs: Ultra-processed foods and beverages
U.S.: United States of America
USD: United States dollars
