# Supplementary material for: What is the purpose of ultra-processed food? An exploratory analysis of the financialisation of ultra-processed food corporations and implications for public health
Source: Global Health. 2023 Nov 13;19:85. doi: 10.1186/s12992-023-00990-1 (PMC10644600; doi:10.1186/s12992-023-00990-1)
Supplement: Supplementary file 1 — Supplementary Material 1 [file 12992_2023_990_MOESM1_ESM.docx]

**Supplementary file 1.** Glossary of key terms

| **Term** | **Definition/description** |
| --- | --- |
| Financialisation | The ‘*increasing role of financial motives, financial markets, financial actors and financial institutions’* in the economy [1]. |
| Hedge fund activists | Hedge fund managers that purchase a minority stake in a publicly listed corporation in order to influence the way in which it is governed [2]. Hedge fund activists very often seek to maximise their returns in the short-term. |
| Market capitalisation | Market capitalisation is calculated by multiplying a corporation’s share price by the number of shares it has outstanding. Market capitalisation represents the monetary value of shareholder wealth stored in a corporation in the form of corporate shares. |
| ‘Real economy’ versus the ‘financial economy’ | Real economy: the section of the economy concerned with the production, trade and consumption or use of goods and services.  Financial economy: the section of the economy that solely deals in transactions involving money and other financial assets. |
| Refranchising | For fast food restaurants, refranchising entails selling company-owned restaurants to franchisees. |
| Share buybacks | Share buybacks refer to the practice of a corporation buying back its own shares on the open stock market. Share buybacks are a means of transferring money from the ‘real economy’ to shareholders. They can also influence financial metrics commonly linked to executive remuneration [3]. |
| Shareholder primacy | The belief or view that the sole purpose of the business corporation should be to maximise returns for its shareholders or private owners because this is the most rational and efficient way of achieving the broader social good [4] |
| Shareholder proposal | A proposed recommendation or requirement made by a shareholder, or group of shareholders, that the corporation or its board of directors take a specific course of action that is put to vote at shareholder meetings [5]. |
| ‘Shareholder value ratio’ | The ‘shareholder value ratio’ is calculated by dividing the total monetary value of shareholder payouts by company revenue, a proxy for the available funds that corporations can distribute among all of its different ‘stakeholders’, including workers, shareholders, governments, and suppliers [6]. |
| Stakeholder capitalism | The World Economic Forum defines stakeholder capitalism as a ‘form of capitalism in which companies seek long-term value creation by taking into account the needs of all their stakeholders, and society at large’ [7]. |
| Total shareholder payouts | Total shareholder payouts can be calculated by adding dividend payments and share buyback expenditure. |
| Ultra-processed foods | Ultra-processed foods (UPFs) are ‘*formulations of ingredients, mostly of exclusive industrial use, that result from a series of industrial processes*’ [8]. Common examples of UPFs include carbonated soft drinks, industrially made snacks and breads, reconstituted meat products, ice creams, confectionery, and many types of breakfast cereals. |
| U.S. listed corporation | A corporation listed on a stock exchange based in the United States of America. |

**References**

1. Epstein G. Financialization and the World Economy. Cheltenham: Edward Elgar Publishing; 2005.

2. DesJardine MR, Marti E, Durand R. Why Activist Hedge Funds Target Socially Responsible Firms: The Reaction Costs of Signaling Corporate Social Responsibility. Academy of Management Journal. 2021;64:851-72. doi: 10.5465/amj.2019.0238.

3. Mazzucato M. Financialization of the Real Economy. The Value of Everything: Making and Taking in the Global Economy. New York: PublicAffairs; 2018.

4. Sjäfell B, Taylor M. Clash of Norms: Shareholder Primacy vs. Sustainable Corporate Purpose. International and Comparative Corporate Law Journal. 2019;13:40-66.

5. Robinson E, Carey R, Foerster A, Sacks G. Latest Trends in Investing for Improved Nutrition and Obesity Prevention. Curr Nutr Rep. 2022. doi: 10.1007/s13668-021-00389-7.

6. Hager SB, Baines J. The Tax Advantage of Big Business: How the Structure of Corporate Taxation Fuels Concentration and Inequality. Politics & Society. 2020;48:275-305.

7. World Economic Forum. What is stakeholder capitalism? 2021 [31 October 2023]. Available from: <https://www.weforum.org/agenda/2021/01/klaus-schwab-on-what-is-stakeholder-capitalism-history-relevance/>.

8. Monteiro CA, Cannon G, Levy RB, Moubarac JC, Louzada ML, Rauber F, et al. Ultra-processed foods: what they are and how to identify them. Public Health Nutr. 2019;22:936-41. doi: 10.1017/S1368980018003762.
