# Supplementary material for: What is the purpose of ultra-processed food? An exploratory analysis of the financialisation of ultra-processed food corporations and implications for public health
Source: Global Health. 2023 Nov 13;19:85. doi: 10.1186/s12992-023-00990-1 (PMC10644600; doi:10.1186/s12992-023-00990-1)
Supplement: Supplementary file 2 — Supplementary Material 2 [file 12992_2023_990_MOESM2_ESM.docx]

**Supplementary file 2.** An overview of the Global Industry Classification Standard (GICS) and the North American Industry Classification System (NAICS), as well as how these were used to inform the categorisation of corporations into five sectors.

The Global Industry Classification Standard (GICS) and North American Industry Classification System (NAICS) are industry analysis frameworks that categorise companies according to their primary economic activities [1, 2].

GICS categorises companies into sectors (two digits), industry groups (four digits), industries (six digits), and sub-industries (eight digits). For example: Consumer staples (30) 🡪 Food, Beverage & Tobacco (3020) 🡪 Beverages (302010) 🡪 Soft drinks and non-alcoholic beverages (30201030). In comparison, NAICS categorises companies into sectors (two digits), subsectors (three digits), industry groups (four digits), industries (five digits), and national industries (six digits). For example: Manufacturing (31-33) 🡪 Beverage Manufacturing (3121) 🡪 Soft drink manufacturing (312112).

In this study, we used the following GICS and NAICS codes to categorise U.S. listed companies into five separate sectors:

| **Sector** | **GICS** | **NAICS (used if differentiation required)** |
| --- | --- | --- |
| Agricultural inputs | 20106015  15101030  15101050^*^ |  |
| Food production, primary processing, and commodity trading | 30202010^**^  30202030^**^ | 111 (excluding 11191, 11192, 11194) or 112 or 114 or 3112 (excluding 31123) or 3113 (excluding 31134, 31135, and major chocolate confectionery manufacturers) or 3115 or 3116 or 3117 or 31192 |
| UPF manufacturing | 30201030  30202010^**^  30202030^**^ | 311 or 31123 or 3133 or 31134 or 31135 or 3114 or 3115 or 3118 or 3119 (excluding 31192) or 312 or 3121 (excluding 312112, 312113, 31212, 31213, and 31214). |
| Food retailing | 30101020  30101030  30101040^**^ | 445 (excluding 4453) or 452^*^ |
| Food service | 25301040 |  |

^*^If the company was identified as being linked with food and agriculture

^**^If the company belonged to one of the corresponding NAICS codes

Companies in the ‘agriculture and farm machinery’ and ‘fertilisers and agricultural chemicals’ sub-industries (GICS) were placed in the agricultural input sector, along with companies in the ‘specialty chemicals’ sub-industry (GICS) with identified links with food and agriculture. Companies in the ‘food distributors’ and ‘food retail’ sub-industries (GICS) were placed in the food retail sector. Companies in the ‘consumer staples merchandise retail’ sub-industry (GICS) were also assigned to the food retail sector if they met one of the following criteria: i) they were in the ‘food and beverage stores’ subsector (NAICS), excluding the ‘beer, wine, and liquor stores’ industry (NAICS); or ii) they belonged to the ‘general merchandise stores’ subsector (NAICS) with identified food retail operations. Companies in the ‘restaurants’ sub-industry (GICS) were grouped in the food service sector.

Categorising companies into the food production, primary processing, and commodity trading sector and the UPF manufacturing sector required more detailed cross-matching between the GICS and NAICS. To start with, corporations in the ‘soft drinks and non-alcoholic beverages’ were moved directly into the UPF manufacturing sector. Next, corporations in the ‘agricultural products and services’ and ‘packaged foods and meats’ sub-industries (GICS) were identified. We then moved corporations in the NAICS groups that related to food and non-alcoholic beverage manufacturing into the UPF manufacturing sector. This was based on our assumption that UPFs are mostly made by food manufacturing corporations (including those under contract with food retailers and restaurants), and that a large proportion of the products that food manufacturing industries produce would most likely be UPFs. Following this, we moved corporations in the NAICS groups that related to food production, primary processing and commodity trading into the food production, primary processing, and commodity trading sector. NAICS, however, does not always completely differentiate industries that produce mostly UPFs from those that produce mostly non-UPFs. As an example, the animal slaughtering and processing, seafood product preparation and packaging, and grain and oilseed milling industries under NAICS are all considered part of the food manufacturing sector. We opted, though, to take a conservative approach by moving these industries out of the UPF manufacturing sector and into the food production, primary processing, and commodity trading sector. As another example, the sugar and confectionery product manufacturing industry under NAICS included both sugar manufacturing, as well as chocolate and confectionery product manufacturing. In this study, we opted move corporations involved in sugar manufacturing into the food production, primary processing, and commodity trading sector, and corporations involved in chocolate and confectionery product manufacturing into the UPF manufacturing sector.

**References**

1. MSCI. The Global Industry Classification Standard (GICS) 2023 [5 September 2023]. Available from: <https://www.msci.com/our-solutions/indexes/gics>.

2. NAICS Association. NAICS Identification Tools 2023 [5 September 2023]. Available from: <https://www.naics.com/search/>.
