# Supplementary material for: What is the purpose of ultra-processed food? An exploratory analysis of the financialisation of ultra-processed food corporations and implications for public health
Source: Global Health. 2023 Nov 13;19:85. doi: 10.1186/s12992-023-00990-1 (PMC10644600; doi:10.1186/s12992-023-00990-1)
Supplement: Supplementary file 3 — Supplementary Material 3 [file 12992_2023_990_MOESM3_ESM.docx]

**Supplementary file 3.** Corporations selected for company-level analysis

We identified four corporations that had consistently held top positions in terms of sector share by revenue, between 1981 and 2021, in the food manufacturing sector and the food service sector (see below). Corporations that have been highly dependent on ultra-processed foods to generate their revenue and profits for most of their histories are highlighted in yellow.

| **Sector** | **Corporation** |
| --- | --- |
| Food manufacturing | Nestlé  PepsiCo  Unilever  Coca-Cola Co |
| Food service | McDonald’s  Starbucks  Sodexo  Yum Brands |
